# Impact of Contextual Factors on the Effect of Interventions to Improve Health Worker Performance in Sub-Saharan Africa: Review of Randomised Clinical Trials

**DOI:** 10.1371/journal.pone.0145206

**Published:** 2016-01-05

**Authors:** Claire Blacklock, Daniela C. Gonçalves Bradley, Sharon Mickan, Merlin Willcox, Nia Roberts, Anna Bergström, David Mant

**Affiliations:** 1 Nuffield Department of Primary Care Health Sciences, University of Oxford, Oxford, United Kingdom; 2 Nuffield Centre for International Health and Development, University of Leeds, Leeds, United Kingdom; 3 Nuffield Department of Population Health, University of Oxford, Oxford, United Kingdom; 4 Gold Coast Health and Griffith University, Queensland, Australia; 5 Nuffield Department of Population Health and Bodleian Healthcare Library, Knowledge Centre, University of Oxford, Oxford, United Kingdom; 6 International Maternal and Child Health, Department of Women’s and Children’s Health, Uppsala University, Uppsala, Sweden; 7 Centre for International Health and Development, Institute of Child Health, University College London, London, United Kingdom; Liverpool School of Tropical Medicine, UNITED KINGDOM

## Abstract

**Background:**

Africa bears 24% of the global burden of disease but has only 3% of the world’s health workers. Substantial variation in health worker performance adds to the negative impact of this significant shortfall. We therefore sought to identify interventions implemented in sub-Saharan African aiming to improve health worker performance and the contextual factors likely to influence local effectiveness.

**Methods and Findings:**

A systematic search for randomised controlled trials of interventions to improve health worker performance undertaken in sub-Saharan Africa identified 41 eligible trials. Data were extracted to define the interventions’ components, calculate the absolute improvement in performance achieved, and document the likelihood of bias. Within-study variability in effect was extracted where reported. Statements about contextual factors likely to have modified effect were subjected to thematic analysis. Interventions to improve health worker performance can be very effective. Two of the three trials assessing mortality impact showed significant reductions in death rates (age<5 case fatality 5% versus 10%, p<0.01; maternal in-hospital mortality 6.8/1000 versus 10.3/1000; p<0.05). Eight of twelve trials focusing on prescribing had a statistically significant positive effect, achieving an absolute improvement varying from 9% to 48%. However, reported range of improvement between centres within trials varied substantially, in many cases exceeding the mean effect. Nine contextual themes were identified as modifiers of intervention effect across studies; most frequently cited were supply-line failures, inadequate supervision or management, and failure to follow-up training interventions with ongoing support, in addition to staff turnover.

**Conclusions:**

Interventions to improve performance of existing staff and service quality have the potential to improve patient care in underserved settings. But in order to implement interventions effectively, policy makers need to understand and address the contextual factors which can contribute to differences in local effect. Researchers therefore must recognise the importance of reporting how context may modify effect size.

## Introduction

Africa bears 24% of the global burden of disease but has only 3% of the world’s health workers [1]. This relative shortage will not be corrected without a redistribution of global economic resources and human capital. However, quality of medical care produced by existing staff varies substantially between facilities [2]. Addressing the substantial variation in care quality produced by existing health workers may be a more feasible immediate-term solution. Robust evidence about which interventions to improve health worker performance are likely to be most effective is required for implementation. By health worker performance, we mean the effectiveness with which existing health workers perform their professional tasks, as measured by their demonstrated skill (e.g. to tie a surgical knot), their care quality (e.g. adherence to clinical guidelines), or the impact of their care (e.g. case-fatality rate).

An overview of strategies to maintain and improve health worker performance in low and middle income countries was previously published [3], but a subsequent paper recognised that the evidence to support policy-making is weak [4]. We were motivated to undertake the current review because our own research (on the shortage of human resources to deliver primary care in sub-Saharan Africa) echoed these findings. It not only suggests that quality of healthcare provision may be of greater importance than quantity of health workers in addressing the inverse care law [5] but also highlighted the difficulty faced by policy makers in selecting evidence-led solutions to improve performance in their local context.

A number of Cochrane reviews address the effectiveness of specific interventions to improve healthcare systems and delivery. While some focus on low and middle-income countries, most include studies conducted in high income western countries and so their applicability to sub-Saharan Africa is questionable [6]. A recent qualitative review applying realist methodology also concluded that the effect of interventions targeted on health worker performance is very context specific, with similar interventions producing very different outcomes when implemented in different circumstances [7].

We have therefore systematically identified trials carried out in sub-Saharan Africa and extracted available data on context, as well as absolute effect, to facilitate the use of the evidence by policy makers. By context, we mean local issues (both within and external to the health care system) reported by authors as having impacted on the effectiveness of the intervention assessed.

## Methods

In conducting the review we adhered to PRISMA guidelines [8]. The aim was to identify trials meeting the following criteria: participants were existing formal health workers; intervention aimed to improve performance; undertaken in sub-Saharan Africa; randomised controlled (RCT) design. Studies recruiting solely health workers in training (e.g. nursing students), informal health workers (e.g. traditional birth attendants), and reported only in conference abstracts were excluded.

**Fig 1:**
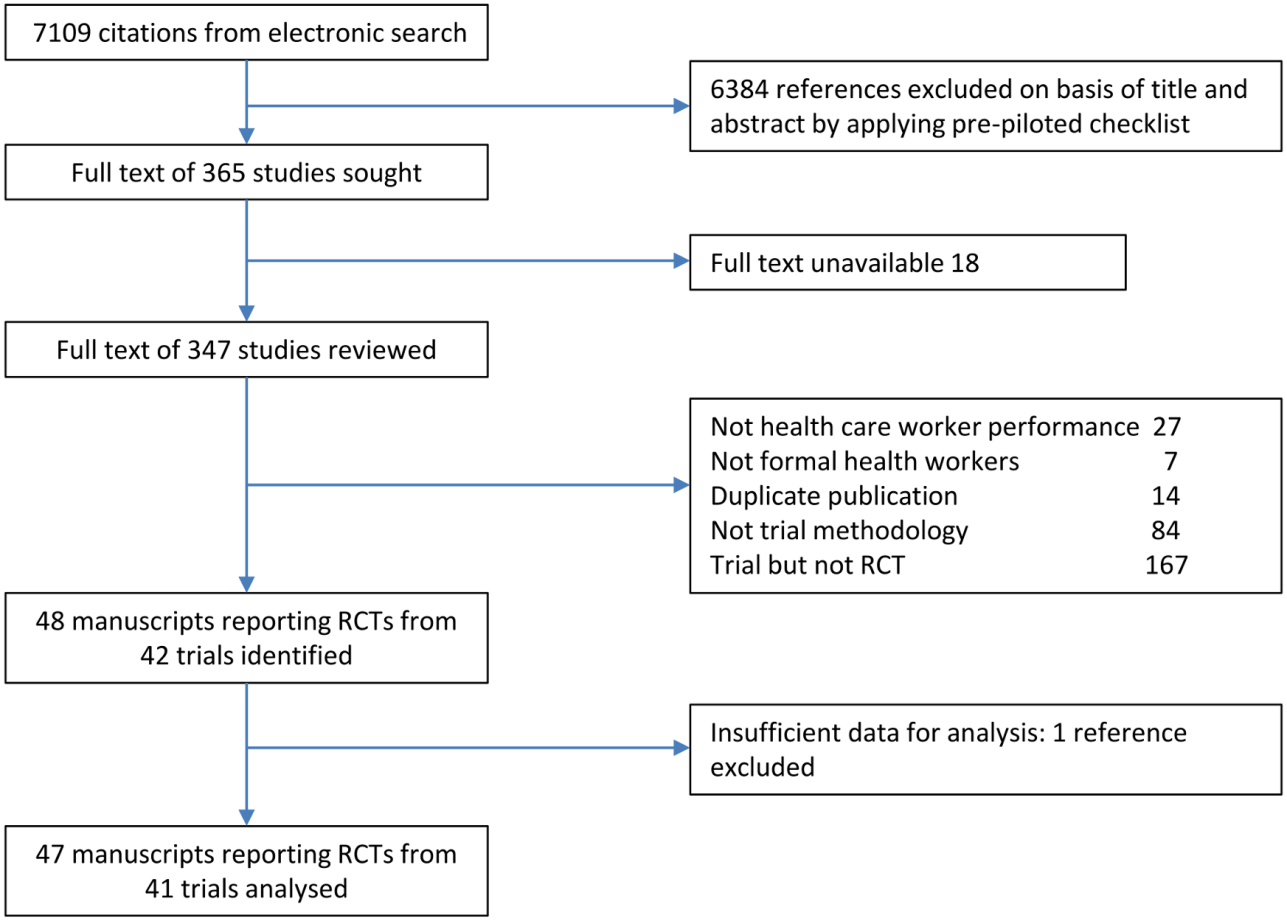
Flow chart of included and excluded studies.

The initial electronic search is detailed in S1 Table. The titles and abstracts of the 7109 papers identified by the electronic search were reviewed by two authors (CB, DGB) who selected 365 for full text review, assessing eligibility for inclusion against a pre-piloted checklist. Disagreements were resolved by discussion after joint re-review of appropriate manuscripts. The flow chart in Fig 1 gives further detail of this review process. As a final step, papers selected were compared with those registered on the database of trials of interventions to improve health worker performance in low and middle income countries compiled by Rowe et al [9].

The Cochrane EPOC Group classification of interventions was used to categorise the components of each intervention [10]. Risk of bias was also assessed against Cochrane criteria [11]. The extent to which interventions had been customised to local circumstances was assessed against two criteria: whether the intervention had been designed or adapted in response to prior assessment of local circumstance, and whether the intervention was based on a specified theory and mechanism for achieving change.

A small number of trials were multi-arm, comparing a number of interventions, often cumulative (i.e. each extra intervention arm including the interventions in other arms). For these studies we report the comparison between the most and least intensive intervention. Many of the studies reported more than one outcome. In selecting the outcome to report in this analysis we applied the following criteria: the stated main objective of the study, the objectivity of the measurement of performance (e.g. observed performance was preferred to self-reported), and the anticipated impact (on performance or health). Three authors (CB, DM, SM) independently identified the outcomes which they felt most closely reflected the stated objective and, if more than one, the most serious outcome in terms of impact. Initial inter-rater agreement in this assessment was high (35/41 studies complete agreement). If outcome at more than one time point was reported, we used the last time point in reporting effect.

In line with our intention to make our findings useful to policy makers, we reported and defined four unique measures. The baseline performance is the pre-intervention performance in the intervention arm (or, if not measured, the performance in the control arm). The absolute change in the intervention group is the difference in the pre- and post-intervention performance in the intervention arm. The absolute difference between the change in the intervention group and the change in the control group is the difference in pre-and post-intervention performance or, if not measured, the difference in end performance. The within study range of change is the minimum and maximum change in performance in the different study sites where reported (or, if not measured, the treatment success rate in the best and worst performing centres in the intervention group).

Extracted data were entered and analysed using Microsoft Excel (2010). The statistical significance reported is the 2-tailed p-value calculated by the authors of the original paper to assess the likelihood that the observed difference in performance between the intervention and control arms could have arisen by chance. The precise value given therefore reflects the way in which the authors chose to report the chosen outcome (e.g. as an absolute difference, risk ratio or odds ratio) and so is an approximation of the statistical significance of the difference between baseline and absolute improvement in performance reported in the tables. However, in all but two trials this p-value was adjusted for clustering and co-variance when appropriate. If the authors reported only confidence intervals, the p-value was calculated from the standard error implicit in the reported intervals (assuming the log of the standard error of an odds ratio is normally distributed).

Contextual effect modifiers were identified when authors incidentally reported factors beyond the intervention, which may have led to differences in outcome between randomisation units (mainly health facilities) within the trial, or contributed to the overall reported trial effect size. Data were identified from the results or discussion text of the reports. In five instances where study authors referred to qualitative publications, they were accessed to confirm and provide additional contextual data. For one identified trial, a post-hoc analysis report was also accessed [12], and related reports were accessed for two further trials [13,14]. The identified context modifiers were analysed by inductive content analysis [15]. Initially three authors (CB, SM and DGB) read each paper thoroughly to become familiar with each author’s language. Through reading and coding, they identified commonality of meaning within and between papers. Through this process of constant comparison, key themes emerged. Higher organisational categories represented a final level of synthesis that facilitated meaningful reporting.

## Results

We report the characteristics of trials, the main trial outcome data, and the contextual data reported by authors. We then present a qualitative analysis of themes emerging from the extracted contextual data.

### Descriptive analysis

The 42 manuscripts reporting the 41 eligible trials identified are shown in Table 1 [16–57]. Two of the included manuscripts reported the same trial (Pirkle [45] and Dumont [28]); however each manuscript reported different aspects of the intervention and different outcomes (one assessing the impact of multi-factorial training on obstetric skills and the other assessing the effect on maternal mortality of focusing training on the lessons learned from maternal death audit). We therefore decided to include both of these manuscripts in this review.

**Table 1:** Characteristics of the selected trials.

First Author	Publication date	Setting	Country	Intervention	EPOC elements^1^	Customised ^2^	
						Change theory	Adapted
Alexander [16]	2013	Private clinics	Kenya	Brief diagnostic training	a,b		x
Autry [17]	2013	Hospital	Uganda	Internet video training	j		
Awad [18]	2006	Health centres	Sudan	Personal discussion of audit	a,b,c,d,g		x
Ayieko [19]	2011	Hospital	Kenya	Training + ongoing supervision	b,c,d,e,g	x	x
Bachmann [20]	2010	Health centres	South Africa	Educational outreach programme	a,b,c,d		
Basinga [21]	2011	Health centres	Rwanda	Financial incentive through pay-for-performance	j		x
Baumgartner [22]	2012	Family planning clinics	South Africa	Training to use decision tools	a,b		x
Bexell [23]	1996	Health centres	Zambia	Training seminars	b		
Biai [24]	2007	Hospital	Guinea-Bissau	Staff financial incentive to facilitate QI programme	g,j		
Bjorkman [25]	2009	Public community clinics	Uganda	Community empowerment project	c,j		x
Brown [26]	2007	Maternity services	South Africa	Multi-dimensional educational package	a,b,h,i		
Buchanan [27]	2014	Health centres	South Africa	Interactive educational model on evidence-based practice	a,b,h		x
Dumont [28]	2013	Hospitals	Mali & Senegal	Training to implement lessons from maternal death audit	b,c,d,g		x
Fairall [29]	2005	Health centres	South Africa	Educational outreach programme	a,b,c,d,h,j		
Gilroy [30]	2004	Health centres	Mali	Training in integrated management of childhood illness	a,b,d,h		x
Grosskurth [31]	1995	Health centres; dispensaries	Tanzania	New STD clinic + staff and community education	b,d,j		
Harrison [32]	2000	Health centres	South Africa	Training and supervision in case management	b,c,d		
Jennings [33]	2010	Health centres	Benin	Counselling training based on communication tools	a,b,c,d,h		
Kalua [34]	2014	Health centres	Kenya, Malawi, Tanzania	Enhanced supervision	b,d	x	x
Kauye [35]	2014	Health centres	Malawi	Diagnostic training	b		
Larke [36]	2010	Health centres	Tanzania	Training in “youth -friendly” service provision	b,d,j		
Lewin [37]	2005	PC centres for TB care	South Africa	Experiential in-service training	a,b,c,d	x	x
Liambila [38]	2010	Private dispensaries	Kenya	Detailing and educational materials	a,d,h		
Loevinsohn [39]	1992	Urban health centres	Sudan	More accessible site and referral for vaccination	j		
Mbacham [40]	2014	Health centres	Cameroon	Training on malaria treatment guidelines	a,b,h		x
Mbonye [41]	2014	Health centres	Uganda	On-site supervision sessions	c,d,g	x	
Meyer [42]	2001	Health centres	South Africa	Training workshops for effective prescribing	b		x
Opiyo [43]	2013	Maternity hospital	Kenya	Presentation of evidence in different ways	a		
Pattinson [44]	2005	Hospitals	South Africa	Educational package to promote KMC	d	x	x
Pirkle [45]	2013	Referral hospitals	Mali & Senegal	Multi-factorial training and supervision package	b,d,e,g		
Rawson [46]	2013	Addiction treatment centres	South Africa	High v low intensity CBT training	b,d,j		
Reynolds [47]	2008	Health centres	Kenya	Quality improvement strategy	a,b,d,h		x
Skarbinski [48]	2009	Government health facilities at all levels	Kenya	Rapid diagnostic test (RDT)	j		
Stanback [49]	2007	Family planning centres	Kenya	Training and supervision	a,b,d		
Steyn [50]	2013	Health centres	South Africa	Use of structured record	a,b,d,h		x
Thiam [51]	2007	Health centres	Senegal	Multifactorial including DOT strategy and supervision	b,d,j		x
Trap [52]	2001	Health centres	Zimbabwe	Stock management protocol	c,d,g		
Weaver [53]	2012	Health centres	Uganda	On-site IMID supervision	d		
Were [54]	2013	Paediatric HIV clinic	Kenya	Computerised reminders	h		
Zurovac [55]	2011	Health centres	Kenya	Text-message reminders	h		
Zwarenstein [56]	2007	Health centres	South Africa	Brief educational outreach training	a,d,h		
Zwarenstein [57]	2011	HIV clinics	South Africa	Educational outreach on HIV care	d		

Notes:

^1^. **EPOC elements**: Components of the intervention based on the Cochrane EPOC Group taxonomy: a) Educational materials; b) Educational meeting; c) Local consensus or Marketing; d) Educational outreach visits; e) Local opinion leaders; f) Patient mediated interventions; g) Audit and feedback; h) Reminders; i) Mass media; j) Other.

^2^. **Customised**: Was any attempt made to customise the intervention through prior assessment of local circumstance (**Adapted**) or through development of a theoretical model of how change would be achieved (**Theory**).

**Abbreviations**: CBT: Cognitive-behaviour therapy; DOT: Direct observation of therapy; IMCI: Integrated management of childhood illness; IMID: Integrated management of infectious disease; KMC: Kangaroo mother care; O&G Obstetrics and gynaecology; PAL: Practical approach to lung health; PC: Primary care; QI: Quality improvement; STD: Sexually transmittable disease; TB: tuberculosis.

Although seven trials were hospital-based, the majority of trials were conducted in primary care facilities, including some private clinics or dispensaries and some specialist clinics (for TB, HIV and STD, family planning and addiction). The analysis of the elements of the intervention according to the EPOC classification shows that just over half the interventions (23/41) were multi-faceted, including three or more elements. The most frequent elements were educational material and meetings. About one-third (17/41) of the trials reported some form of prior assessment of local context to customise the intervention, but only in five was a formal theory of change presented. Five of the trials referred to a total of six accompanying qualitative papers in their discussions, of which four were available for analysis [58–61]. We also accessed a post-hoc analysis report [12] relating to one trial [19], and additional reports [13,14] related to a further two trials [35,33].

**Fig 2:**
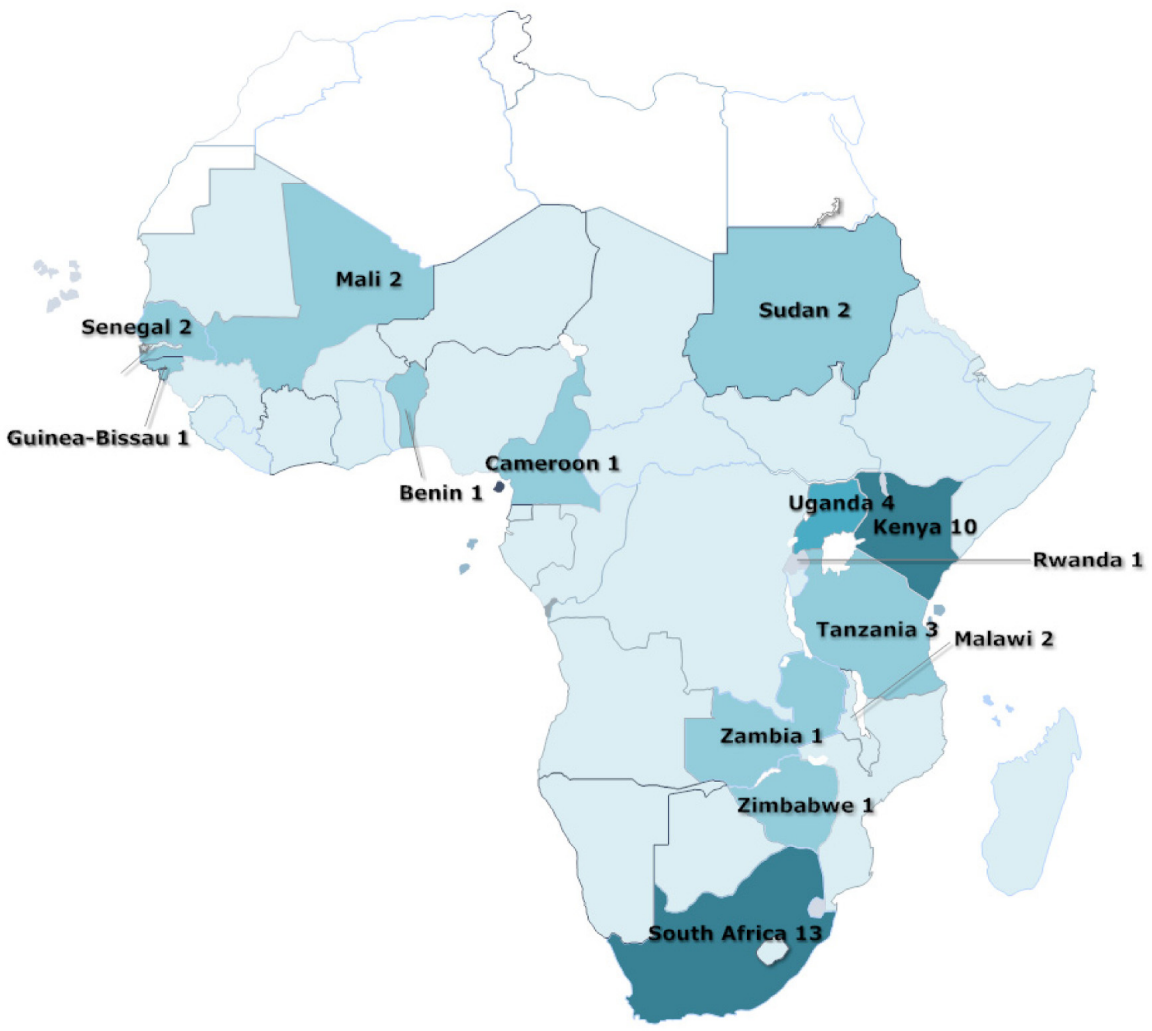
Sub-Saharan countries in which the trials identified had been undertaken. Reprinted from SmartDraw Software LLC under a CC BY license, with permission from SmartDraw Software LLC, original copyright 2013.

**Fig 3:**
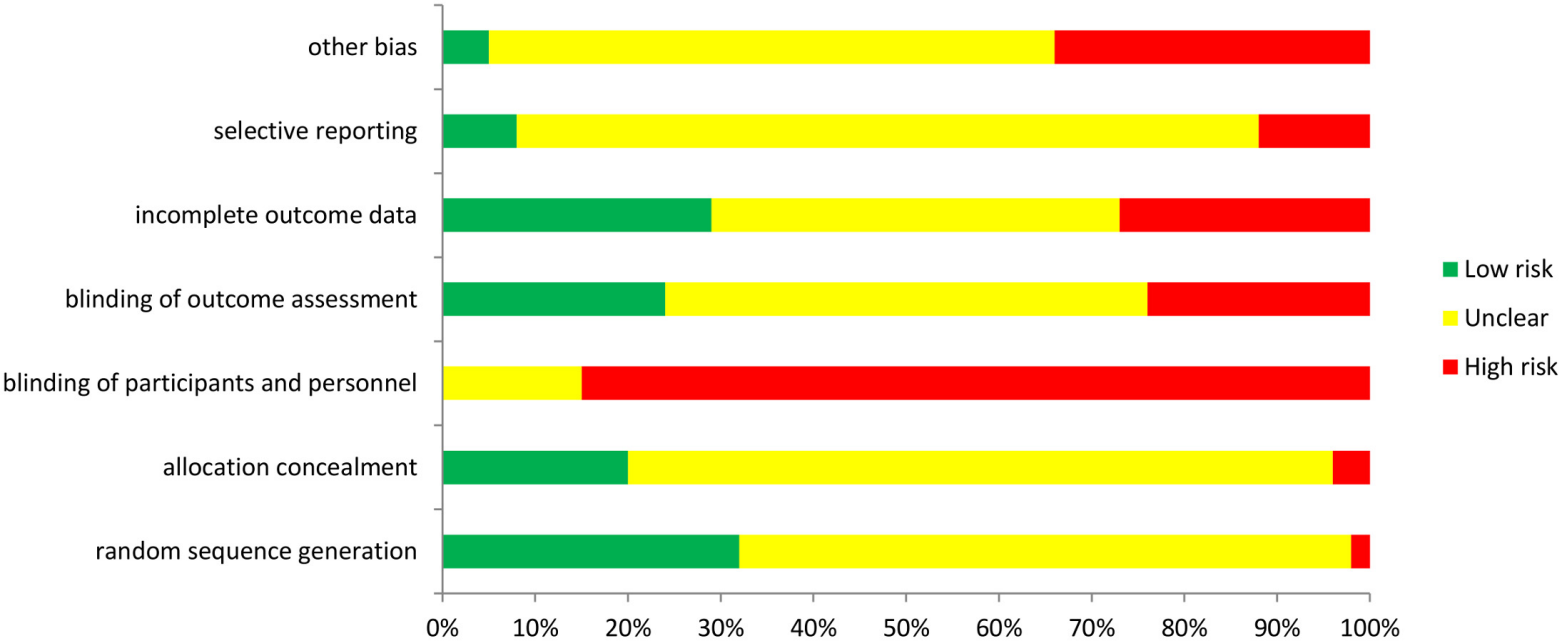
Risk of bias assessed against Cochrane criteria.

Half of the trials (23/41) were conducted in South Africa or Kenya with most of the rest in other East African countries (Fig 2). Only eight trials were conducted in West Africa (one trial had sites in both Mali and Senegal [28]) and one in Central Africa, in Cameroon [40]. Fig 3 shows the overall assessment of risk of bias. In many cases it was difficult or impossible to make assessment of outcome blinded to the intervention arm and most trials were incompletely reported.

### Effect of interventions aimed at improving skills (Table 2)

**Table 2:** Interventions to improve health service management and clinical skills: change in performance indicators, and contextual effect modifiers.

Author	Outcome	Baseline or control performance^1^	Absolute change in the intervention group^2^	Absolute difference between the change in the intervention group and the change in the control group^3^	p-value	Key contextual effect modifiers	Supporting evidence for contextual modifiers
***Improvement in health service management***							
Reynolds	Health workers report being observed by supervisor	31%	+22%	+26%	<0.01	Supervisory staff turnover, Functionality of local facilities (e.g. electricity)	Author comments
Trap	Drug availability	73%	+7%	+10%	ns	Staff ability and motivation	Author refers to ability to calculate minimum stock reported Table 1
***Improvement in clinical skills***							
Alexander	Ability to diagnose depression	84%	-	+1%	ns	Patient and provider lack of resources (e.g. medication), Patient resistance to diagnosis of mental illness, Staff basic training, Language and cultural acceptance of mental illness	Author refers to survey data
Autry	Quality score of surgical knot tying improved by at least 50%	14%	-	+61%	<0.05	Internet speed, Unfamiliarity of local surgeons with technique (not usually used in Uganda to conserve sutures) may have disadvantaged control group	Author comments
Buchanan	Evidence-based practice knowledge score (% max score)	47%	+23%	+7%	ns	Poor acceptability of intervention, Low baseline knowledge	Author comments and refers to attrition data
Gilroy	IMCI counselling quality mean score	26%	-	+8%	<0.01	Staff previous training, basic ability, and willingness to learn, Language used in consultation, Workload constraints (volume of patients, possibility for privacy), Language of training resources	Quantitative influence of consultation language on effect of intervention presented in Fig 1
Kalua	Eye care knowledge and skills score (% max score)	50%	+15%	+12%	<0.01	Staff turnover and vacant positions, Absenteeism	Author comments and presents turnover and absentee data
Kauye	Diagnostic sensitivity for depression	3%	-	+57%	<0.001	Extent to which training adapted to local context	Author comments
Opiyo	Neonatal care clinical knowledge score	70%	-	-8%	ns	Baseline skills in evidence-based medicine, Extent to which participants had gone through pre-workshop materials	Qualitative data presented
Rawson	Cognitive Behavioural Therapy (CBT) skills mean score	49%	+7%	+6%	<0.01	Staff turnover	Dropout data presented
Stanback	Family planning self-reported practice mean score	49%	+23%	+8%	<0.001		
Weaver	IMID clinical knowledge mean score	50%	-1%	-1%	ns	English language skills, Motivation of participants/ fatigue	Author refers to data showing statistically significant effect of assessment fatigue

Notes:

^1^. **Baseline or control performance** is the pre-intervention performance in the intervention arm, or if not measured, the end performance in the control arm.

^2^. **Absolute change in the intervention group** is the absolute difference in the pre- and post-intervention performance of the intervention arm.

^3^. **Absolute difference between the change in the intervention group and the change in the control group** is the difference between the change in pre-and post-intervention performance in intervention and control arms, or if not measured, the difference in end performance between intervention and control arms.

IMCI, integrated management of childhood illness; IMID, integrated management of infectious disease

There was substantial variation in the effect of these interventions (Table 2); for example, one intervention aimed at improving diagnosis of depression in Kenya was associated with no improvement [16] while another in Malawi reported a 20-fold improvement in diagnostic sensitivity from 3% to 57% (p<0.001) [35]. Other interventions with substantial effect sizes were internet-video based training to improve ability to tie surgical knots (61% greater improvement in the intervention group compared to control, p<0.05, though local suture-conserving techniques influenced outcome) [17] and a management intervention in Kenya to encourage supervisors to improve their effectiveness (increase in proportion directly observing staff performance from 31% to 53%, p<0.01) [47].

The most frequent contextual factors cited by authors as influencing outcome were staff motivation, supervision and turnover. None of these studies reported the within-study heterogeneity between centres.

### Effect of interventions to improve the quality of the care process (Table 3)

**Table 3:** Interventions to improve prescribing and other treatment processes: change in performance indicators, and contextual effect modifiers.

Author	Outcome	Baseline or control performance^1^	Absolute change in the intervention group^2^	Absolute difference between the change in the intervention group and the change in the control group^3^	p-value	Key contextual effect modifiers	Supporting evidence for contextual modifiers	Within study range of change^4^
***Improvement in prescribing***								
Awad	Inappropriate antibiotic prescribing	25%	-22%	-20%	<0.001	Staff basic training and supervision. Patient expectations	Author comments	-
Ayieko	Correct IV fluid prescribing	7%	+60%	+30%	<0.01	Staff basic training, induction training, staff turnover and personal motivation. Adequacy of management, supervision, informal training.	Author comments. Data presented for turnover. 2x qualitative publications cited.	+46% to +77%^b^
Baumgartner	Re-injection of depot contraception	68%	-	+26%	<0.01	Local “stock-outs” of drugs	Outlier intervention clinic presented	-
Bexell	Correct drug choice	52%	+26%	+17%	<0.05	Adequacy of staff supervision and support to develop new routines. Local “stock-outs” of drugs	Author comments	-
Harrison	Correct STD treatment	36%	+52%	+48%	<0.01	Local “stock-outs” of drugs and other supplies (e.g. condoms, information cards). Motivation of staff. Community perceptions about quality of care	Author comments	-
Liambila	Provision of emergency contraception	85%	+6%	-1%	ns	Local workload (no time available to deliver intervention). High staff turn-over, Cultural acceptance of discussing sexually transmitted disease	Author comments and refers to qualitative data	-
Meyer	Correct prescribing for diarrhoea and vomiting	31%	+16%	+15%	<0.05	None reported		-
Mbacham	Adherence to malaria guidelines	37%	-	+18%	ns	In-facility training	Author comments	-5% to +35%^a^
Mbonye	Appropriate malaria treatment	46%	+31%	+23%	ns	Belief in reliability/accuracy of results	Author comments	-40% to +70%^b^
Skarbinski	Recommended antimalarial (A-L) given for uncomplicated malaria over 5 years	59%	-23%	-63%	0.04	Clinical competence in ordering test for malaria. Local “stock-outs” of drugs	Author comments. Author refers to data in Table 4	-
Zurovac	Correct management of malaria using A-L	21%	+31%	+25%	<0.01			-
Zwarestein 11	Provision of co-trimoxazole prophylaxis	32%	-	+9%	<0.05	Customisation of training to perceived local need, Local “ceiling effects” (variable room for improvement)	Author comments and refers to qualitative data. Qualitative publication cited.	-
***Improvement in other treatment processes***								-
Brown	% Deliveries with childbirth companion	9%	+12%	-1%	ns	Local management. High staff turnover. Lack of resources. Workload.	Author comments	0% to 40%^c^
Basinga	Institutional deliveries	35%	-	+8%	<0.05	Adequacy of monitoring and supervision. Adequacy of financial incentive to outweigh local implementation barrier	Author comments	-
Fairall	TB detection	4%	-	+3%	<0.05	Managerial support provided by educational trainers. Perceived salience of intervention by individual staff	Author comments and refers to baseline data Table 1	-
Jennings	Antenatal care (% issues communicated)	51%	+17%	+20%	<0.01	Local workload and facilities (no time and space available to deliver intervention). Language barriers	Author refers to qualitative data	-
Larke	Clinic attendance	458/month	+121/month	+56/month	ns	High staff turnover. Local “stock-outs” of condoms	Author comments. Qualitative publication cited.	-
Loevinsohn	Childhood vaccination rate	54%	+32%	+2%	ns	Degree of close supervision of staff, Failure of parents to bring vaccination cards	Author comments	-
Pattinson	KMC implementation median score	38%	-	+14%	<0.05	Hospital management structures	Heterogeneity of results between hospitals	-33% to +61%^a^
Pirkle	Clinical audit (obstetrics) mean score	65%	-	+3%	<0.05	Resource constraints (workload and equipment to complete tasks). Leadership and ownership of changes	Author refers to between-country differences in data	-
Were	Completion of clinical tasks (HIV care)	18%	-	+50%	<0.001	Completeness and quality of local recording of clinical data	Author refers to survey results	-

Notes:

^1^. **Baseline or control performance** is the pre-intervention performance in the intervention arm, or if not measured, the end performance in the control arm.

^2^. **Absolute change in the intervention group** is the absolute difference in the pre- and post-intervention performance of the intervention arm.

^3^. **Absolute difference between the change in the intervention group and the change in the control group** is the difference between the change in pre-and post-intervention performance in intervention and control arms, or if not measured, the difference in end performance between intervention and control arms.

^4^. **Within study range of change** is range of difference between intervention and control endpoints at different study sites^a^, range of change at different intervention facilities^b^, or range of endpoint at different intervention facilities^c^.

IV, intravenous; STD, sexually transmitted disease; A-L, artemether-lumefantrine; TB, tuberculosis; KMC, Kangaroo Mother Care; HIV, human immunodeficiency virus

There was again substantial variation in effect (Table 3), ranging from no impact of an intervention in a South African hospital to promote childbirth companions, as an incentive to improve the performance of obstetric staff [26], to a Kenyan study showing an improvement in correct IV fluid prescribing in intervention paediatric wards of 30%, compared to controls (p<0.01) [19]. All but two [38,48] of the interventions to improve prescribing were effective in achieving a clinically important improvement, most (8/12) showing a statistically significant effect compared to control, ranging from 9 to 48%.

Five of the studies reported within study heterogeneity. Where the range of difference between paired intervention and control sites was reported [40,44], this exceeded mean effect. The most frequent contextual factor cited as influencing within-study effect were stock-outs of drugs and adequacy of supervision. High staff turnover was also mentioned as a contextual modifier in four trials and personal motivation in two.

### Effect of interventions to improve impact of care (treatment outcomes) (Table 4)

**Table 4:** Interventions to improve treatment outcomes: change in performance indicators, and contextual effect modifiers.

Author	Outcome	Baseline or control performance^1^	Absolute change in the intervention group^2^	Absolute difference between the change in the intervention group and the change in the control group^3^	p-value	Key contextual effect modifiers	Supporting evidence for contextual modifiers	Within study range of change^4^
***Improvement in treatment outcome***								
Bachmann	Successful TB treatment	64%	-	+4%	ns	None stated		-
Biai	<5 case-fatality from malaria	10%	-	-5%	<0.01	Adequacy of supervision and post-training supportLack of personal motivation (attributed to inadequate pay)	Author comments	-
Bjorkman	<5 deaths/1000 live births (under 5 mortality rate)	144/1000	-	-47/1000	ns	Degree of community participation, Extent of role taken by local community facilitators	Author refers to data Fig 3	-0.9 to +0.2 standard deviations^c^
Dumont	Maternal deaths/1000 patients (crude in-hospital maternal mortality rate)	10.3/1000	-3.5/1000	-2.5/1000	<0.05	Local leadership and ownership, Local resource constraints, Affordability of Caesarean sections (local donor-sponsored programme)	Author comments and refers to presented data	-7.6 to +0.4/1000^a^
Grosskurth	HIV seroconversion	1.90%	-	-0.7%	<0.01	Low acceptability and use of condoms	Author comments	-0.3% to -1.2%^a^
Lewin	Successful TB treatment completion	54%	+6%	+3%	ns	Motivation of local clinic manager, Lack of agency to change fixed work patterns, High staff turnover, staff conflict and poor teamwork	Author comments. Qualitative paper cited (unavailable)	-18% to +32%^b^
Steyn	Uncontrolled diabetes (HbA1c >7%)	63%	+2%	+2%	ns	Staff shortages, Lack of necessary equipment (e.g. to do blood tests)	Authors refers to qualitative data	-
Thiam	Successful TB treatment	68%	+20%	+12%	<0.05	Adequacy of local supervision, Local “stock-outs” of drugs	Author comments	82.3% to 94.5%^c^
Zwarestein 07	Asthma severity mean score	86%	-45%	-9%	<0.05	None stated	Qualitative paper cited (unavailable)	-

Notes:

^1^. **Baseline or control performance** is the pre-intervention performance in the intervention arm, or if not measured, the end performance in the control arm.

^2^. **Absolute change in the intervention group** is the absolute difference in the pre- and post-intervention performance of the intervention arm.

^3^. **Absolute difference between the change in the intervention group and the change in the control group** is the difference between the change in pre-and post-intervention performance in intervention and control arms, or if not measured, the difference in end performance between intervention and control arms.

^4^. **Within study range of change** is range of difference between intervention and control endpoints at different study sites^a^, range of change at different intervention facilities^b^, or range of endpoint at different intervention facilities^c^. TB, tuberculosis; <5, under 5 years; HIV, human immunodeficiency virus; HbA1c, glycated haemoglobin

Two interventions to improve health worker performance achieved important reductions in mortality (Table 4)–in child mortality (case fatality age<5 years reduced from 10% to 5%, p<0.01, in Guinea-Bissau [24]) and in-hospital maternal mortality (from 10.3/1000 to 6.8/1000, p<0.05, in Mali/Senegal [28]). Increasing the involvement of the community in monitoring local health services was also associated with a substantial reduction in <5 child mortality (from 144 to 97/1000) but the statistical power of the trial was very limited as even an effect of this magnitude was non-significant [25].

The within-study variability was reported for five trials. Where reported, the range in the difference between intervention and control sites in different strata exceeded mean effect [28,31]. The most frequently cited contextual modifiers were again drug stock-outs and supervision / continuing in-service support. The three trials with the greatest within-study range of improvement all cited the quality of local management and leadership as key issues.

### Thematic analysis of context

**Fig 4:**
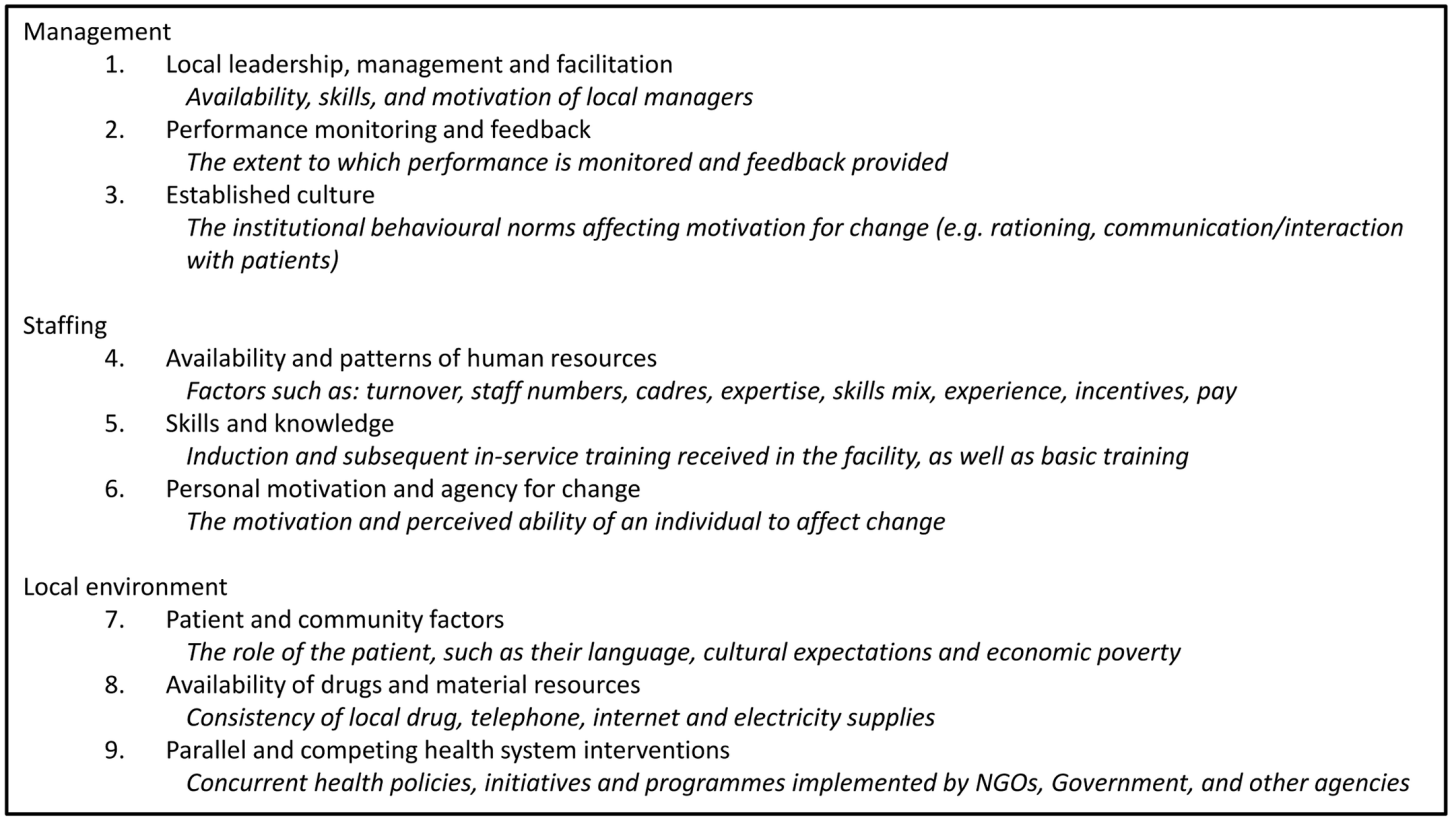
Thematic analysis of contextual effect modifiers.

The results of the thematic analysis of contextual effect modifiers are summarised in Fig 4, and included in full in S2 Table with illustrative quotes. Nine unique themes have been synthesised into three organisational headings for ease of understanding: management, staffing, and local environment. We have provided detailed quotes for each of the nine key themes, within the higher order organisational headings. This illustrates the depth of meaning for each contextual theme as reported by each trial (see S2 Table: Thematic analysis of contextual effect modifiers, for direct quotations from the trials).

#### Management

The management theme which emerged most frequently was the effectiveness of local leadership. In more than one case, the lack of leadership was attributed to the frequent rotation of supervisory staff. When interventions failed to achieve the intended improvement in performance, it was frequently attributed to lack of subsequent local supervision, support or follow up (“*…inadequately supported training*, *supervision and follow-up*, *which has resulted in their infrequent use by the prescribers”* [18]). The other strong management theme was organisational inertia, poor team-working and resistance to change (“*…poor team working and staff conflicts were common; task-orientated care was entrenched”* [37]).

#### Staffing

The three staffing themes identified were: the absolute shortage of staff, the adequacy of existing knowledge and skills, and erosion of personal motivation or of perceived personal agency to effect change. The shortage of staff (“*the redistribution of patients to the primary level resulted in increasing patient numbers but this was unaccompanied by increases in staff numbers and clinic facilities”* [50]) was exacerbated by high staff turnover and inadequacy of basic, induction, and follow-up in-service training (“*Training …should be followed up by support and reinforcement”* [24]). A lack of personal motivation to change, and a failure of individuals to see themselves as possible agents for change, was expressed in relation to both supervisors and staff (“*……staff did not see themselves as having the agency to initiate workplace change”* [37].)

#### Local environment

The environmental themes identified related to: material resources, patient and community factors, and concurrent health system interventions. The most frequently cited issue was “stock-outs”, such as failure of the supply chain for drugs (“*Regular availability of drugs is an important factor influencing the credibility*, *confidence and utilization of health services among the population*” [23]) although lack of other material supplies, such as electricity failure or poor telephone/internet reception, was sometimes also an issue. Many patient and community factors (such as poverty) were reported which cannot be easily reformed, but the impact of customising interventions to reflect community issues such as language, cultural expectation and transport issues was widely recognised (“*… [intervention materials] were adapted to the [local] environment and*... *used locally relevant graphics to enhance engagement with low-literate women*” [33]). Finally, concurrent programmes implemented by other agencies (particularly NGOs) were often mentioned as having an important impact on the measured local effect of the trialled intervention (“*The poor effect in [regional] hospitals outside the capital could be due to potential contamination bias*” [28]).

## Discussion

Our review shows three important things. Firstly, it is possible to improve the performance of existing health workers serving in under-resourced health systems. Secondly, the success of an intervention in improving performance depends very much on local context, with differences reported between centres within trials. Thirdly, few trials have been undertaken in sub-Saharan Africa and even fewer have adopted methodologies which allow the effect of local context to be assessed (e.g. only a minority applied a theory of change model or reported difference in effect between participating centres).

The wide range of interventions assessed and outcomes measured in very different settings means that a simple comparison of relative effect has limited policy relevance. However, the positive performance of interventions to improve prescribing in 8/12 included trials is policy relevant (even if this is because it is a straightforward care function to target and measure). The parallel contextual observation that drug “stock-outs” were a frequent cause of variation in the within-study effect also emphasises the policy importance of securing the medicines supply-chain.

The fact that the range of performance improvement exceeded the mean in many trials reporting this information is perhaps the most important observation. However, we are not the first to note the modifying effect of local context on outcome and the limited policy utility of focusing on mean effect. Others have previously stressed that triallists should give better information on contextual effects [62] and tools to facilitate their measurement have been developed applicable to high-income settings [63]. Bonnell [64] and Michie [65] have both suggested mechanisms for better specifying the process by which the intervention achieves performance improvement, allowing the impact of key contextual modifiers to be estimated. The growing emphasis on process evaluation for trials of complex interventions is an important step. Process evaluations can explore intervention theory and the influence of context on its implementation and effect. Data on recruitment, delivery, response, maintenance, effectiveness and unintended consequences, are gathered during and after the trial using mixed methods [66]. The new MRC guidance ‘Process evaluation of complex interventions’, emphasises the need to design and evaluate key contextual components [67]. However, challenges faced by some have included maintaining methodological quality [68], costs, how to integrate evaluation findings with traditional trial outcomes [69], and making communication of findings accessible [66].

The informal way in which contextual data were reported in our included trials weakens the generalisability of our findings while also reflecting the lack of systematic effort to report and understand aspects of context which influence the effect of interventions. We could only extract what was reported incidentally, and this is likely to be incomplete. Despite this, the frequency with which the same contextual issues recurred in different manuscripts is consistent with the existing literature and echoes the context elements of the PARIHS framework [70]. The themes also support the contextual factors perceived to influence the implementation of interventions as measured by the Alberta Context Tool [63] and the Context Assessment for Community Health tool [71]. Thirteen of our included trials were conducted in South Africa. Although South Africa differs in some ways to other sub-Saharan countries, we found that the emerging themes were consistent between countries. Representation of particular countries and settings in this study should be taken into consideration when interpreting the findings.

In order to know what will work locally, and to implement any intervention effectively, policy makers need to understand and address the specific contextual factors which contribute to differences in effect sizes seen in trials. To implement the “knowledge to action cycle” promoted by the Canadian Institute of Health Research, it is essential to identify the local barriers and facilitators likely to impact on the effectiveness of an intervention [72], and to also consider the underlying theory and mechanisms of achieving change [64, 65]. Evidence about key effect modifiers not only informs the choice of intervention, but also facilitates the context to be modified, and the intervention “tailored” [73], as part of the implementation process.

In other words, those who undertake trials need to recognise the importance of providing sufficient evidence to make clear whether intervention X will work in setting Y. Our results show that interventions to improve the performance of existing health workers have the potential to impact very positively on patient morbidity and mortality in an underserved sub-Saharan context. The importance of local contextual influence in intervention trials should be reflected in both trial reporting, and in intervention planning and design.

### Protocol Registration

An initial protocol for the review was registered with PROSPERO 2014:CRD42014007391.

## Supporting Information

S1 Table Electronic Search Strategy. (DOCX)

S2 Table Thematic Analysis of contextual effect modifiers (full). (DOCX)

S3 Table PRISMA Checklist. (DOC)

S1 Text Initial registered protocol. (PDF)

S2 Text Revisions made to registered protocol. (DOCX)
