# Supplementary material for: Impact of Contextual Factors on the Effect of Interventions to Improve Health Worker Performance in Sub-Saharan Africa: Review of Randomised Clinical Trials
Source: PLoS One. 2016 Jan 5;11(1):e0145206. doi: 10.1371/journal.pone.0145206 (PMC4701409; doi:10.1371/journal.pone.0145206)
Supplement: S1 Table — (DOCX) [file pone.0145206.s001.docx]

**S1 Table: Electronic Search Strategy**

We searched the following electronic databases from inception to January 7^th^ 2015, using terms to describe health workers, performance, and interventions, with a filter for low and middle-income countries: CINAHL [EBSCOHost](1982-07/01/2105), Cochrane Central Register of Controlled Trials [Cochrane Library, Wiley](Issue 12, January 2014), EMBASE [OvidSP](05/01/2015), Global Health [OvidSP](1973-07/01/2015), Global Health Library [Virtual Health Library](07/01/2015), Medline & Medline In-process [OvidSP](1946-07/01/2015), PsycINFO [OvidSP](1967-December Week 5 2014). No restrictions were applied for publication date or language. A medical librarian (NR) assisted in the design and conduct of the search.

The search terms applied for Medline are shown below:

| 1 | exp Health Personnel/ |
| --- | --- |
| 2 | ((health* or care* or medical or clinical or nursing or midlevel or mid-level or hospital*) adj2 (professional? or worker? or staff* or officer? or provider?)).ti,ab. |
| 3 | (doctor? or physician? or surgeon? or clinician?).ti,ab. |
| 4 | (nurse? or midwife or midwives or physiotherapist? or therapist? or pharmacist? or technician?).ti,ab. |
| 5 | (assistant? or aide?).ti,ab. |
| 6 | 1 or 2 or 3 or 4 or 5 |
| 7 | exp Hospitals/ |
| 8 | exp Ambulatory Care Facilities/ |
| 9 | ((health or health care or healthcare or medical or ambulatory or clinical) adj2 (centre? or center? or unit? or facility or facilities)).ti,ab. |
| 10 | clinic?.ti,ab. |
| 11 | 7 or 8 or 9 or 10 |
| 12 | 6 or 11 |
| 13 | Developing Countries/ |
| 14 | (Africa or Caribbean or West Indies or South America or Latin America or Central America).hw,kf,ti,ab,cp. |
| 15 | (Afghanistan or Albania or Algeria or Angola or American Samoa or Argentina or Armenia or Armenian or Azerbaijan or Bangladesh or Benin or Byelarus or Byelorussian or Belarus or Belorussian or Belorussia or Belize or Bhutan or Bolivia or Bosnia or Herzegovina or Hercegovina or Botswana or Brazil or Brasil or Bulgaria or Burkina Faso or Burkina Fasso or Upper Volta or Burundi or Urundi or Cambodia or Khmer Republic or Kampuchea or Cameroon or Cameroons or Cameron or Camerons or Cape Verde or Central African Republic or Chad or China or Colombia or Comoros or Comoro Islands or Comores or Mayotte or Congo or Zaire or Costa Rica or Cote d'Ivoire or Ivory Coast or Cuba or Djibouti or French Somaliland or Dominica or Dominican Republic or East Timor or East Timur or Timor Leste or Ecuador or Egypt or United Arab Republic or El Salvador or Eritrea or Ethiopia or Fiji or Gabon or Gabonese Republic or Gambia or Gaza or Georgia Republic or Georgian Republic or Ghana or Gold Coast or Grenada or Guatemala or Guinea or Guinea-Bisau or Guam or Guiana or Guyana or Haiti or Honduras or Hungary or India or Maldives or Indonesia or Iran or Iraq or Jamaica or Jordan or Kazakhstan or Kazakh or Kenya or Kiribati or Korea or Kosovo or Kyrgyzstan or Kirghizia or Kyrgyz Republic or Kirghiz or Kirgizstan or Lao PDR or Laos or Lebanon or Lesotho or Basutoland or Liberia or Libya or Macedonia or Madagascar or Malagasy Republic or Malaysia or Malaya or Malay or Sabah or Sarawak or Malawi or Nyasaland or Mali or Marshall Islands or Mauritania or Mauritius or Agalega Islands or Mexico or Micronesia or Middle East or Moldova or Moldovia or Moldovian or Mongolia or Montenegro or Morocco or Ifni or Mozambique or Myanmar or Myanma or Burma or Namibia or Nepal or Netherlands Antilles or Nicaragua or Niger or Nigeria or Pakistan or Palau or Palestine or Panama or Papua New Guinea or Paraguay or Peru or Philippines or Philipines or Phillipines or Phillippines or Romania or Rumania or Roumania or Rwanda or Ruanda or Saint Lucia or St Lucia or Saint Vincent or St Vincent or Grenadines or Samoa or Samoan Islands or Navigator Island or Navigator Islands or Sao Tome or Senegal or Serbia or Seychelles or Sierra Leone or Sri Lanka or Ceylon or Solomon Islands or Somalia or Sudan or Suriname or Surinam or Swaziland or Syria or Principe or South Sudan or Tajikistan or Tadzhikistan or Tadjikistan or Tadzhik or Tanzania or Thailand or Timor-Leste or Togo or Togolese Republic or Tonga or Tunisia or Turkey or Turkmenistan or Turkmen or Tuvalu or Uganda or Ukraine or Uzbekistan or Uzbek or Vanuatu or New Hebrides or Venezuela or Vietnam or Viet Nam or West Bank or Yemen or Zambia or Zimbabwe or Rhodesia).hw,kf,ti,ab,cp. |
| 16 | ((developing or less* developed or under developed or underdeveloped or middle income or low* income or underserved or under served or deprived or poor*) adj (countr* or nation? or population? or world)).ti,ab. |
| 17 | ((developing or less* developed or under developed or underdeveloped or middle income or low* income) adj (economy or economies)).ti,ab. |
| 18 | (low* adj (gdp or gnp or gross domestic or gross national)).ti,ab. |
| 19 | (low adj3 middle adj3 countr*).ti,ab. |
| 20 | (lmic or lmics or third world or lami countr*).ti,ab. |
| 21 | transitional countr*.ti,ab. |
| 22 | 13 or 14 or 15 or 16 or 17 or 18 or 19 or 20 or 21 |
| 23 | 12 and 22 |
| 24 | exp Health Personnel/ed [Education] |
| 25 | inservice training/ or staff development/ |
| 26 | exp Education, Continuing/ |
| 27 | exp Teaching/ |
| 28 | (educat* or train* or teach* or learn*).ti. |
| 29 | ((educat* or train* or teach* or learn*) adj2 (material? or aid? or tool? or information or guide?)).ti,ab. |
| 30 | ((educat* or train* or teach* or learn*) adj2 intervention?).ti,ab. |
| 31 | (workshop? or seminar? or webinar? or tutorial? or meeting?).ti,ab. |
| 32 | (elearning or e-learning or online learning or online teaching or online training or online module?).ti,ab. |
| 33 | Information dissemination/ |
| 34 | *guidelines as topic/ or *practice guidelines as topic/ |
| 35 | (guideline? or protocol?).ti,ab. |
| 36 | ((written or print*) adj2 (material? or aid? or tool? or information or guide?)).ti,ab. |
| 37 | (handbook? or hand-book? or guidebook? or guide-book?).ti,ab. |
| 38 | Mentors/ |
| 39 | academic detailing.ti,ab. |
| 40 | ((academic or education*) adj2 outreach).ti,ab. |
| 41 | (supervision or supervisor? or mentor* or opinion leader?).ti,ab. |
| 42 | (audit and feedback).ti,ab. |
| 43 | Reminder Systems/ |
| 44 | (reminder? or prompt?).ti,ab. |
| 45 | (decision* adj2 (aid? or tool? or system?)).ti,ab. |
| 46 | ((job or work*) adj2 aid?).ti,ab. |
| 47 | advice.ti,ab. |
| 48 | telephone/ or exp cellular phone/ |
| 49 | Internet/ |
| 50 | (internet* or web* or electronic* or computer* or online).ti. |
| 51 | ((internet* or web* or electronic* or computer* or online) adj3 (support or tool? or aid?)).ti,ab. |
| 52 | ((internet* or web* or electronic* or computer* or online) adj3 intervention?).ti,ab. |
| 53 | (cell phone* or cellphone* or cellular phone* or mobile phone* or iphone* or i-phone* or text messag* or sms).ti,ab. |
| 54 | ((multifacet* or multi-facet*) adj2 intervention*).ti,ab. |
| 55 | 24 or 25 or 26 or 27 or 28 or 29 or 30 or 31 or 32 or 33 or 34 or 35 or 36 or 37 or 38 or 39 or 40 or 41 or 42 or 43 or 44 or 45 or 46 or 47 or 48 or 49 or 50 or 51 or 52 or 53 or 54 |
| 56 | "quality of health care"/ or "outcome and process assessment (health care)"/ or quality assurance, health care/ or quality improvement/ or quality indicators, health care/ |
| 57 | quality.ti. |
| 58 | (quality adj5 (health* or care or indicator* or improve* or manage* or measur* or develop*)).ti,ab. |
| 59 | Guideline Adherence/ |
| 60 | ((guideline* or protocol*) adj5 (adhere* or complian* or comply or implement* or disseminat*)).ti,ab. |
| 61 | professional competence/ or clinical competence/ |
| 62 | Physician's Practice Patterns/ |
| 63 | Employee Performance Appraisal/ |
| 64 | exp Professional Role/ |
| 65 | (performance or competenc* or capacity).ti,ab. |
| 66 | appraisal?.ti,ab. |
| 67 | medical errors/ or diagnostic errors/ or exp medication errors/ |
| 68 | (medical adj2 (error* or mistake?)).ti,ab. |
| 69 | ((medication* or prescri*) adj2 (error* or mistake* or inappropriate)).ti,ab. |
| 70 | ((error? or harm?) adj3 (reduc* or lower* or less*)).ti,ab. |
| 71 | 56 or 57 or 58 or 59 or 60 or 61 or 62 or 63 or 64 or 65 or 66 or 67 or 68 or 69 or 70 |
| 72 | 23 and 55 and 71 |
| 73 | randomized controlled trial.pt. |
| 74 | controlled clinical trial.pt. |
| 75 | randomized.ab. |
| 76 | placebo.ab. |
| 77 | drug therapy.fs. |
| 78 | randomly.ab. |
| 79 | trial.ab. |
| 80 | groups.ab. |
| 81 | 73 or 74 or 75 or 76 or 77 or 78 or 79 or 80 |
| 82 | exp animals/ not humans.sh. |
| 83 | 81 not 82 |
| 84 | 72 and 83 |
| 85 | Intervention Studies/ |
| 86 | intervention?.ti. or (intervention? adj6 (clinician? or collaborat$ or community or complex or DESIGN$ or doctor? or educational or family doctor? or family physician? or family practitioner? or financial or GP or general practice? or hospital? or impact? or improv$ or individuali?e? or individuali?ing or interdisciplin$ or multicomponent or multi-component or multidisciplin$ or multi-disciplin$ or multifacet$ or multi-facet$ or multimodal$ or multi-modal$ or personali?e? or personali?ing or pharmacies or pharmacist? or pharmacy or physician? or practitioner? or prescrib$ or prescription? or primary care or professional$ or provider? or regulatory or regulatory or tailor$ or target$ or team$ or usual care)).ab. |
| 87 | (pre-intervention? or preintervention? or "pre intervention?" or post-intervention? or postintervention? or "post intervention?").ti,ab. |
| 88 | (pre-post or "pre test$" or pretest$ or posttest$ or "post test$" or (pre adj5 post)).ti,ab. |
| 89 | (pre-workshop or post-workshop or (before adj3 workshop) or (after adj3 workshop)).ti,ab. |
| 90 | (before adj10 (after or during)).ti,ab. |
| 91 | (time points adj3 (over or multiple or three or four or five or six or seven or eight or nine or ten or eleven or twelve or month$ or hour? or day? or "more than")).ab. |
| 92 | ("time series" adj2 interrupt$).ti,ab. |
| 93 | 85 or 86 or 87 or 88 or 89 or 90 or 91 or 92 |
| 94 | exp animals/ not humans.sh. |
| 95 | 93 not 94 |
| 96 | 72 and 95 |
| 97 | 96 not 84 |
