# Supplementary material for: Impact of Contextual Factors on the Effect of Interventions to Improve Health Worker Performance in Sub-Saharan Africa: Review of Randomised Clinical Trials
Source: PLoS One. 2016 Jan 5;11(1):e0145206. doi: 10.1371/journal.pone.0145206 (PMC4701409; doi:10.1371/journal.pone.0145206)
Supplement: S2 Table — (DOCX) [file pone.0145206.s002.docx]

**S2 Table: Thematic analysis of contextual effect modifiers**

| **Theme** | **Illustrative quotes** |
| --- | --- |
| **1. Local leadership, management and facilitation**  *Availability, skills, motivation and experience of local managers.*  **Comments**  Mid-level managers and senior ward staff are seen to be key agents for change.  The recognition given to staff by local management appears to be important.  **Implications**  Managers have the power to change aspects of the organisation’s structure and function to positively influence the impact of the intervention.  For example, they can;   - provide time, support for education - supervise, follow up education - ensure adequate staffing - improve service availability - encourage patient monitoring - coordinate expectations - plan, support change - support, supervise staff - implement staff performance feedback - promote team working - extend positive benefits to other services, policy | *[E]ase of implementation will clearly vary according to the structure and functioning of the health services.* (Grosskurth, 1995, p.535)  *Organisational changes to improve 24 hr service availability and patient monitoring contributed most to improving emergency obstetric clinical care and, as a consequence, to improving maternal outcomes.* (Dumont, 2013, p.154)  *We had not appreciated the level of organisation required [to implement the intervention] and the need for substantive shift in organisation by the staff working [on site] [...] It seems that despite a willingness to implement[the intervention] policies, practical restrictions and adverse health service conditions such as transfer of staff [...], militated against success.* (Brown, 2007, p.5)  *However, providing regular support supervision and performance feedback [to support the intervention] are not routine. Resources and systems for supervision need strengthening and supervisors themselves need training and organizing.* (Ayieko, 2011, p.12)  *Our results identified the deficiencies in implementation [of intervention] through inadequately supported training, supervision and follow-up, which has resulted in their infrequent use by the prescribers. [...] Supervision and follow-up are essential to provide good quality of care.* (Awad, 2006, p.141)  *This contrasted with the generally weak clinic and middle management in the trial intervention clinics [...] poor team working and staff conflicts were common [...]* (Lewin, 2005, p.256)  *Failure to replace staff (such as the eye coordinator who was a supervisor) who leave for long periods of time weakens the systems. Weak systems make the provision of quality care, especially specialised care, more challenging, and also make it imperative that good quality supervision take place and include skills transfer*. (Kalua, 2014, p.6)  *…across all the hospitals, there was considerable variation in the role of departmental in-charges, with only a few displaying clear leadership…* (Nzinga 2009 (Ayieko study qualitative report), p.4) |
| **2.** **Performance monitoring and feedback**  *The extent to which performance is monitored and feedback provided*  **Comments**  Many routine information systems are inadequate to measure change and do not provide regular feedback.  **Implications**  Positive, documented and observable change is required to maintain an intervention.  Data needs to be monitored, and human performance supervised to track and maintain intervention integrity.  It is important to engage healthcare communities to accurately collect and report data – Health Care Workers need to understand (and trust) the importance of systematic, reliable and accurate data collection, performed where possible, within normal clinical routines. | *One possible explanation for results favouring the controls is that reporting may be more accurate in intervention clinic, where the intervention has made health workers more aware of the need to record data systematically.* (Harrison, 2000, pp.2777-8)  *Rational health policy decisions depend on reliable data on the cost-effectiveness of different health interventions. (Grosskurth, 1995, p.534)*  *[C]ommunity monitoring can play an important role in improving service delivery when traditional top-down supervision is ineffective [...] It may also be the case that combining bottom-up monitoring with a reformed top-down approach could yield even better results.* (Bjorkman, 2009, p.767)  *[...] increased monitoring and supervision led to increased provider effort.* (Basinga, 2011, p.1246)  *Routine information systems are inadequate to generate the data required to evaluate care and the capacity for conducting and disseminating analyses as part of routine feedback is largely absent.* (Ayieko, 2011, p.12)  *Reporting may be more accurate in intervention clinics, where the intervention has made health workers aware of the need to record data systematically.* (Harrison, 2000, p.2777-2778)  *In applying this tool, one assumes that what is recorded in the medical chart has actually been done and what has not been recorded, has not been done. This assumption may be flawed.* (Pirkle, 2013, p.11)  *[I]n 28% of the clinical encounters where clinicians were given a reminder for an overdue task but did not complete this task at that visit, clinicians reported that the test, medication, or referral had previously been ordered. In these cases, the relevant information was likely not reflected in the [electronic health record], suggesting a breakdown in the system connecting the clinician, patient, laboratory, and [electronic health record].* (Were, 2013, p.e795)  *A system or culture unable to appreciate and recognise work done well was also reported by health workers to be a major barrier to encouraging correct practice, not just for implementing the new guidelines. They complained that there was more emphasis on work done badly, explaining that this was a major cause of loss of morale. (Nzinga 2009 (Ayieko study qualitative report), p.5)* |
| **3. Established culture**  ***T****he entrenched institutional behavioural norms affecting institutional motivation for change (e.g. rationing, communication/ interaction with patients)*  **Comment**  Could be summarised as ‘The way things are done around here’. *^ǂ^  **Implications**  There were many reported differences between individuals which contributed to the variability in process and outcome data.  Noted differences between clinicians, include;   - their abilities - previous training, education levels - willingness to learn - capacity to implement new skills - prescribing patterns - test ordering - communication with patients - charging patients - team working   Ways in which culture can enhance an intervention are;   - Health Care Worker care and attention towards patients can enhance their adherence to advice and increase confidence in the health system | *The extra care and attention given to the patients seemed to influence how well they adhered to the advice of the health personnel and increased their confidence in the health system.* (Biai, 2007, p.5)  *The considerable variation in the average score of those [intervention] trained health care providers could also be affected by their educational level, their willingness to learn in the training sessions or their capacity to implement the newly learned [intervention] skills.* (Gilroy, 2004, p.42)  *Interestingly between-clinician differences contributed almost two-thirds of overall variability in the data, suggesting that hospitals as organisations do not necessarily promote consistent practice amongst clinicians within them.* (Opondo *(Ayieko study post-hoc report)* 2011, p.7)  *In practice, health workers mostly prescribed [the specific drug] only after laboratory confirmation, but did not increase diagnostic test use sufficiently [...] our results showed that we need a better understanding of when and why health workers order diagnostic tests.* (Skarbinski, 2009, p.924)  *[P]oor team working and staff conflicts were common; task-orientated care was entrenched [...]* (Lewin, 2005, p.256)  *There was a steady decline in the intervention hospitals in the capital cities. For the district hospitals outside the capital cities, the benefit was demonstrated later (year 4), following the educational programme [...] with no significant effect for regional hospitals.* (Dumont, 2013, p.151)  *Participants were not documenting information related to patient interventions sufficiently to be able to evaluate the effects of their interventions* (Buchanan, 2014, p.8)  *In fact, in general all cadres rarely discussed mistakes made by colleagues, reporting that they avoid unnecessary confrontations by making corrections, but not following the mistake through to its source* (Nzinga 2009 (Ayieko study qualitative report), p.6)  *Several comments also pointed to inter-cadre conflicts that may be considerable barriers to dissemination and uptake of new practices* (Nzinga 2009 (Ayieko study qualitative report),  p.5)  *Senior or older clinicians were often reported to be stuck in the patterns of previous practice, although there were also exceptions to this observation.* (Nzinga 2009 (Ayieko study qualitative report), p.6) |

| **4. Availability of human resource**  *Factors such as: turnover, low numbers, cadres, expertise, skills mix, experience, incentives, low pay*  **Comment**  These workforce factors are sometimes innate (e.g. absolute lack of trained health workers) but often dynamic (e.g. rapid turnover due to unsustainable workloads and lack of support)  **Implications**  Managers can improve the following staffing aspects;   - support and encourage new routines - maintain adequate staffing for clinical facilities - maintain, clarify, expand job roles - allocate staff for training - identify appropriately skilled staff to implement intervention   When poorly managed, health system reform and restructuring can be demotivating, encourage staff turnover  There are also documented incentives for staff;   - financial incentives - specific targets - support from peers - travel to meetings   The relationship between incentives and performance is not always directly linear. Incentives may be more powerful when providers have control over their work | *The redistribution of patients to the primary level resulted in increasing patient numbers, but this was unaccompanied by increases in staff numbers and clinic facilities.* (Steyn, 2013, p.7)  *…and characteristics of the individual health providers, such as innate counseling abilities, and/or previous training, likely contributed to the sizable variation between [community health centres]. The considerable variation in the average scores of those IMCI-trained health care providers could also be affected by their educational level, their willingness to learn in the training sessions, or their capacity to implement the newly learned counseling skills.* (Gilroy, 2004, p.42)  *The increase in caseload in the intervention clinics was caused mainly by changes in the district's largest clinic, perhaps related to increased staff motivation after the intervention, or by more rapidly changing community perceptions of improved quality of care*. (Harrison, 2000, p.2777)  *Even a small financial incentive and a specific target [...] can make workers more likely to adhere to standards and improve the quality of their work.* (Biai, 2007, p.5)  *[H]igher payments provide stronger incentives [...] incentives have a larger effect on services in which providers have more control over delivery [...] the incentive payment gave providers the motivation to translate their knowledge [about the topic] into better practice.* (Basinga, 2011, p.1247)  *[H]ealth system reform and restructuring, which have increased turnover of experienced staff and exacerbated uncertainty among those who remain.* (Lewin, 2005, p.257)  *[O]ne alternative may be to explore the feasibility and effectiveness of expanding the role of less skilled health workers who may have fewer time constraints.* (Jennings, 2010, p.11)  *The practice of transferring in-charges will reduce the effectiveness of any training intervention unless the in-charges take their skills to their new facilities and mechanisms are in place to train the new supervisors.* (Reynolds, 2008, p.65)  *There was a high turnover of PHCWs, with only 20% of the workers interviewed at baseline still in place at the end of 2 years; the positions of most of those who had left had been filled by others*. (Kalua, 2014, p.5)  *There was also a high absenteeism with approximately 1/3 of all PHCWs absent during the two visits*. (Kalua, 2014, p.5)  *Improving the skills of general HWs has been shown to be challenged by high levels of attrition, absenteeism, and turn over in other settings in Africa, findings which are reflected in our study*. (Okwen 2014 (Kalua study single country report), p.99) |
| --- | --- |
| **5. Skills and knowledge**  *Reflecting the adequacy of induction and subsequent in-service training received the facility, as well as basic training*  **Comment**  Basic training may be limited, or even a barrier to best practice, so adequacy of induction training is important.  One-off training is not enough - ongoing supervision and in-service training is necessary.  Coverage of new training amongst staff is important, particularly if there is high staff turnover, or poor institutional cascade of information.  **Implications**  It is widely recommended to create integrated educational packages that include;   - complementary components - local adaptation, prioritisation - high quality research evidence - sustained intensity, frequency of training in the workplace - wide distribution of trained staff - systematic continuing education - online simulation activities - monitoring, reinforcement, supervision of what is learned (knowledge, skills) - audit & feedback strategies to monitor progress - documented clinically important changes in processes of care | *[V]ery high turnover of staff at health facilities [...]. Ideally all health facility staff would receive [induction] training.* (Larke, 2010, p.520)  *Another recommended approach is to increase post-training supervision in health centres [...]* (Gilroy, 2004, p.42)  *[The intervention programme] uses workshops to allow labour staff to identify changes in practice they regard as important, set goals for change, and to use an audit and feedback mechanism to monitor progress.* (Brown, 2007, p.7)  *Training is one of the main activities of health programmes, but it should be followed up by support and reinforcement.* (Biai, 2007, p.5)  *[N]eed to integrate their educational activities in a more systematic primary health care continuing education program.* (Bexell, 1996, p.357)  *Simulation allows for repetitive and deliberate practice, rather than observatory learning. [...] The interns in remote teaching group self reported practicing more [...] because they knew they would be evaluated [...]* (Autry, 2013, p.130)  *[T]raining programmes that have been adapted to the local context are likely to be more effective than generic ones. [...] The training sessions were also based on what each country considered as priorities in their country.* (Kauye, 2013, p.665)  *The main source of treatment information in such practices is likely to be from drug company representatives.* (Zwarenstein, 2007, p.7)  *[This] trial suggests that where baseline knowledge is low, any mode of education may make a difference* (Buchanan, 2014, p.7)  *Many of the clinicians surveyed did not attend the training workshops and therefore the knowledge and practice of those treating patients in whom the outcomes were measured was most likely informed by the in-facility training, or no training. This limits our ability to estimate the effect of attending workshops compared with participating in in-facility training*. (Mbacham, 2014, p.355)  *The fact that even after training, only 35% of HWs could accurately carry out a visual acuity test suggests that training was too short in duration, not taught adequately, or was too complex for these HWs*. (Okwen 2014 (*Kalua study single country report*),p.99) |
| **6. Personal motivation and agency for change**  *The motivation and perceived ability of an individual to effect change.*  **Comment**  This may be affected by trust/confidence in advice, perceived usefulness/preferences etc.  **Implications**  The skills and desire to facilitate change can enhance the implementation and sustainability of an intervention. Change requires personal and organisational effort. These skills must usually be learned, by individuals and by managers. They are best developed over time, in a respectful supervisory relationship. This supervisory relationship can mediate between difficult policies, limited material resources and clinical challenges.  It is more likely that individuals will change their behaviour when changes;   - happen in direct care tasks - are locally monitored - identify impact on other service areas   However, without supervision, staff can quickly feel angry, frustrated, despondent and ultimately choose not to adhere to what they know should be done. | *[P]oor or slow uptake may be associated with a requirement for greater personal or organisational effort to change, the view that a task is not directly related to care of the immediate illness, or, in intervention sites, an area unlikely to be subject to local evaluation.* (Ayieko, 2011, pp.11-12)  *To make the staff accept corrections and changes in behaviour after supervision, they need to be interested and available, otherwise neither the supervisor nor the training would have any effect.* (Biai, 2007, p.5)  *[Intervention] training generally resulted in more respectful, informative and less judgemental consultations with [target population].* (Larke, 2010, p.519)  *As the staff felt these recommendations were idealistic and not feasible in their demanding work situation, this induced a sense of frustration, alienation, and in certain cases rejection of the guidelines.* (Steyn, 2013, pp.7 & 9)  *For health workers to make the calculation requires maths ability [...] as well as the motivation through understanding how the calculation will be used.* (Trap, 2001, p.278)  *[A]nd staff did not see themselves as having the agency to initiate workplace change.* (Lewin, 2005, p.256)  *The estimates showed larger effects on services for which facilities receive larger financial incentives and those over which the provider has greater control [...] and are less dependent on patients’ health-seeking behaviour.* (Basinga, 2011, p.1245)  *The fact that one third of the eligible participants declined to be enrolled in the trial reflects on the acceptability of the intervention and the generalisability of the reported results to this target group.* (Buchanan, 2014, p.10)  *Convincing the clinicians to believe in laboratory results was an uphill task.* (Mbonye, 2014, p.14)  *…some [health facilities] may not have always had a complete stock, especially if health workers were involved in illegal re-selling of drugs and supplies, as was sometimes reported.* (Plummer 2006 (Larke qualitative paper),p.463)  *In their interviews, all nurses emphasized the high emotional toll of their ever-increasing workload with HIV positive patients who, at this early stage of the roll-out, are generally very ill* (Stein 2008 (Zwarenstein qualitative paper), p.5-6) |

| **7. Patient and community factors**  *The role of the patient, such as their language, cultural expectations and economic poverty, in constraining the performance of health personnel*  **Comment**  Important examples raised as contextual factors were: community poverty, language, educational and cultural barriers, accessibility to transport, ability to purchase prescribed medication, adherence with prescribed care.  **Implications**  Patients and the community also have the power to change the way they seek and access health care, which can positively influence the impact of the intervention.  Positive health- seeking behaviour can be influenced by;   - greater awareness of care - choices of care - use of local language, graphics, customs - (perceptions of) improved quality of care - continuity of care, longer term management - widespread dispersal of HCW working throughout community   The community impact takes a longer time (than management led change) and is influenced by many other cultural, health system and gender issues. | *Changes in health-seeking behaviour may depend on a recognition that the quality of care has improved, and on efforts to raise awareness and encourage prompt care-seeking.* (Harrison, 2000, p.2777)  *Pressure from patients on doctors to prescribe [drugs] even in the absence of an appropriate indication [...] patients’ expectations for treatment should be considered to limit the overuse of [drugs].* (Awad, 2006, p.141)  *This increase in attendance was likely to reflect health-seeking behaviour.* (Larke, 2010, p.519)  *One potential factor is that the [intervention materials] were adapted to the [local] environment and were easy to use [...] used locally relevant graphics to enhance engagement of low-literate women.* (Jennings, 2010, p.10)  *Such findings highlight the importance of quality assessments to identify gaps and improve services, including assessing new mothers’ cultural perceptions as a factor in newborn care*. (Jennings, 2014, pp.563-4)  *Barriers in access to care and other social and economic factors may undermine women's ability to introduce and maintain practices at home despite increased awareness to do so*. (Jennings, 2014, p.564)  *Most villagers walked 3-10 kilometres to visit [a health facility]* (Plummer 2006 (Larke qualitative paper), p.463)  *Adults condemned young people’s out-of-wedlock sexual relationships and severely punished girls who were caught (Wight et al. in press), so secrecy about young people’s STIs was great.* (Plummer 2006 (Larke qualitative paper), p.463) |
| --- | --- |

| **8. Lack of drugs and material resources**  *Limitations posed by constraints of material resources such as local drug “stock-outs” and electricity failure*  **Comment**  A recurring theme was lack of regular supply of drugs, but shortage of equipment, inadequate facilities (such as lack of electricity, water or working toilets) and lack of internet/ phone coverage also influenced local implementation  **Implications**  Material resources need careful planning and budgeting to complement the intervention. Often a lack of resources limits the integrity and impact of an intervention.  Important resources to consider are;   - reliable availability of drugs, equipment - sufficient space, patient privacy - use of multiple languages - dedicated time, in addition to normal care - sufficient laboratory space, blood banks   In planning change, it is important to adapt to the resources available, and to begin with procedures that require least additional resources and time for their implementation. | *Practice change was consistently more likely for procedures that could be stopped easily [...] and less likely for procedures that required additional resources or time to implement.* (Brown, 2007, p.7)  *Regular availability of drugs is an important factor influencing the credibility, confidence and utilization of health services among the population.* (Bexell, 1996, p.357)  *[St]ock-outs would be more likely outside a trial setting and may reduce facility use substantially.* (Larke, 2010, p.519)  *[La]ck of or limited dedicated space [for delivering the intervention] remained a challenge, along with language barriers.* (Jennings, 2010, p.11)  *This finding reinforces the important distinction between treatment and management, and highlights the importance of improving the accessibility of [appropriate] drugs at the primary care level [...]* (Harrison, 2000, p.2777)  *Characteristics of the [setting], such as high adult patient volume and privacy of the [intervention] room [..] likely contributed to the sizable variation between [settings].* (Gilroy, 2004, p.42)  *Potentially, [drug] rationing and prescriber confusion could have been minimised by guaranteeing an adequate and stable supply of [drug] while completely withdrawing non-recommended [drugs].* (Skarbinski, 2009, p.925)  *The on-site, in-charge supervisors may not be able to remedy broader systems-related problems such as lack of electricity, waiting room conditions or working toilets. However, results associated with examination area conditions in training groups were more promising, particularly with aspects such as having more water.* (Reynolds, 2008, p.65)  *Resource limitations, particularly in [one of the settings], may be slightly more influential in determining the care of these patients (especially those with direct complications) than interventions targeting medical practice.* (Pirkle, 2013, p.11)  *Although the [intervention] made recommendations that constituted good clinical practice, they did not take budgetary restraints or the contexts in which the staff were working into consideration.* (Steyn, 2013, p.7)  *[P]rovider counselling and maternal reporting were lowest in these categories [postnatal visits and family planning] perhaps due to low contraceptive uptake and limited family planning services in the region*. (Jennings, 2014, p.564)  *[A]ll supervisory visits to the intervention facilities occurred as scheduled, except for two visits in Malawi which were not made due to unavailability of fuel.* (Kalua, 2014, p.5) |
| --- | --- |
| **9. Parallel and competing health system interventions**  *The local effect of concurrent health policies, initiatives and programmes implemented by NGOs, Government, and other agencies.*  **Comment**  Conflicting or concurrent health policies, initiatives and programmes may positively or negatively impact on the implementation of interventions, depending on whether their priorities conflict with or complement those of the intervention.  **Implications**  It is important not to develop strategy to improve health worker performance without awareness of external factors likely to impact on factors like staff motivation and retention.  The integration of government-funded horizontal programmes with donor-funded vertical programmes is essential. | *The poor effect in [regional] hospitals outside the capital could be due to potential contamination bias. Indeed, during the period of this trial, international (bilateral cooperation) and governmental organisations implemented [similar components to the intervention programme] in four of the seven regional hospitals in the control group.* (Dumont, 2013, p.154)  *Whereas condoms now seem to be relatively well-accepted in Mwanza town, partly due to the intensive condom promotion activities there, acceptability and use of condoms remain low in rural areas….*(Grosskurth, 1995, p.535)  *[The national control program] benefited from two large scale, on-going [continuous quality improvement] programs, which would have confounded the measurement of the effects of [the current intervention].* (Weaver, 2012, p.8)  *The trial was conducted at a time of major restructuring of the health services in [the country].* (Steyn, 2013, p.7)  *Another possible concern is that the [intervention] was implemented in the context of a larger health sector reform.* (Basinga, 2011, p.1426)  *This research was also conducted among intervention and control sites who were participating in a QI initiative at the time of the study* (Jennings, 2014, p.564)  *[P]ublic health system did improve in a short period of strong economic growth over this period*. (Muinga, 2014, p.7)  *[L]ocal pharmaceutical industry representatives were able to influence the choice of drugs so that clinicians ignored the guidelines*. (Nzinga 2009 (Ayieko study qualitative report), p.7) |

^*^McCormack B, Kitson A, Harvey G, Rycroft-Malone J, Titchen A, Seers K. *Getting evidence into practice: The meaning of 'context'.* J Adv Nurs. 2002;38(1):94-104.

^ǂ^Drennan D. *Transforming company culture*. 1992, London: McGraw-Hill.
