# Supplementary material for: Impact of Contextual Factors on the Effect of Interventions to Improve Health Worker Performance in Sub-Saharan Africa: Review of Randomised Clinical Trials
Source: PLoS One. 2016 Jan 5;11(1):e0145206. doi: 10.1371/journal.pone.0145206 (PMC4701409; doi:10.1371/journal.pone.0145206)
Supplement: S1 Text — (PDF) [file pone.0145206.s004.pdf]

UNIVERSITY of York
Centre for Reviews and Dissemination

NHS
National Institute for
Health Research

PROSPERO International prospective register of systematic reviews

Which interventions improve health worker performance in low and middle-income countries?

Claire Blacklock, Daniela Goncalves, Merlin Willcox, David Mant

Citation

Claire Blacklock, Daniela Goncalves, Merlin Willcox, David Mant. Which interventions improve health worker performance in low and middle-income countries?. PROSPERO 2014:CRD42014007391 Available from http://www.crd.york.ac.uk/PROSPERO_REBRANDING/display_record.asp?ID=CRD42014007391

Review question(s)

What is the effectiveness of interventions to improve health worker performance in low and middle-income countries?

Which methods for assessing health worker performance are used to measure the effect of intervention studies in low and middle-income countries?

Searches

CINAHL, Cochrane Central Register of Controlled Trials (CENTRAL), EMBASE, Global Health, Medline, PsycINFO

Types of study to be included

Included:

RCTs, non-randomised intervention studies with pre-and post-intervention measurements.

Excluded:

Reviews, protocols only, cross-sectional descriptive studies.

Condition or domain being studied

Implementation research

Health systems

Participants/ population

Inclusion criteria:

Health workers in LMIC (a person who has the title of a health worker or is a professional employed within the health service - can be any cadre including lay health workers, traditional healers, and can extend to professional health service administrators e.g. health managers)

Exclude:

Population not health workers in LMIC, e.g. interventions targeted at patient behaviour/adherence/access to treatment rather than health worker performance, not in LMIC

Intervention(s), exposure(s)

Inclusion:

Intervention to improve health worker performance or related patient outcome measure (includes theoretical knowledge, clinical skills, communication skills/ethics)

Exclude:

Not intervention studies, e.g. review articles, cross-sectional descriptive studies, published clinical guidelines, study protocols

Comparator(s)/ control
Non-exposed group if study has control group.

Outcome(s)
Primary outcomes
Change in health worker performance, or related patient outcome.

Secondary outcomes
Method of measuring performance.

Data extraction, (selection and coding)
Two researchers will independently review all identified references from the search for inclusion in the review. Any disagreements will be resolved by discussion, or by a third researcher if required.

Data to be extracted (not finalised):

• Setting: country and health facility

• Health worker: cadre, number of participants

• Intervention: describe (later categorise, e.g. written material, training workshop etc.), duration, cost

• Study type: e.g. RCT

• Method used to assess performance: describe (later categorise e.g. prescribing, observation etc.)

• Effect of intervention (including time after completion)

Risk of bias (quality) assessment
Quality will be assessed using accepted standard checklists. The synthesis will take quality of study into account.

Strategy for data synthesis
Where possible, a quantitative synthesis of aggregate data will be performed and reported.

Analysis of subgroups or subsets
Planned subgroup analyses:

Type of intervention

Type of health worker

Method of assessing performance

Contact details for further information
Claire Blacklock

2nd Floor, New Radcliffe House, Jericho

Oxford

claire.blacklock@phc.ox.ac.uk

Organisational affiliation of the review
Nuffield Department of Primary Care Health Sciences, University of Oxford

www.phc.ox.ac.uk

Review team
Dr Claire Blacklock, Nuffield Department of Primary Care Health Sciences
Dr Daniela Goncalves, Nuffield Department of Primary Care Health Sciences
Mr Merlin Willcox, Nuffield Department of Primary Care Health Sciences
Professor David Mant, Nuffield Department of Primary Care Health Sciences

Anticipated or actual start date
29 January 2014

Anticipated completion date
20 June 2014

Funding sources/sponsors
The Nuffield Department of Primary Care Health Sciences, University of Oxford receives funding from the NIHR SPCR.

Conflicts of interest
None known

Language
English

Country
England

Subject index terms status
Subject indexing assigned by CRD

Subject index terms
Allied Health Personnel; Developing Countries; Health Personnel; Humans; Job Satisfaction

Stage of review
Ongoing

Date of registration in PROSPERO
29 January 2014

Date of publication of this revision
29 January 2014

Stage of review at time of this submission	Started	Completed
Preliminary searches	No	Yes
Piloting of the study selection process	No	Yes
Formal screening of search results against eligibility criteria	Yes	No
Data extraction	No	No
Risk of bias (quality) assessment	No	No
Data analysis	No	No

The information in this record has been provided by the named contact for this review. CRD has accepted this information in good faith and registered the review in PROSPERO. CRD bears no responsibility or liability for the content of this registration record, any associated files or external websites.
