# Supplementary material for: Impact of Contextual Factors on the Effect of Interventions to Improve Health Worker Performance in Sub-Saharan Africa: Review of Randomised Clinical Trials
Source: PLoS One. 2016 Jan 5;11(1):e0145206. doi: 10.1371/journal.pone.0145206 (PMC4701409; doi:10.1371/journal.pone.0145206)
Supplement: S2 Text — (DOCX) [file pone.0145206.s005.docx]

**Revisions made to registered protocol**

We registered an initial review protocol with PROSPERO in January 2014 (2014:CRD42014007391). (Please see the attached PDF copy of this protocol.) The registered protocol was for a larger review, to include other study types, in addition to RCTs.

Given the quantity of studies identified from the search for the larger review, we decided to narrow our inclusion criteria for the analysis presented in the submitted paper, to:

- existing formal health workers
- randomised controlled (RCT) design

Studies recruiting solely health workers in training (e.g. nursing students), or informal health workers (e.g. traditional birth attendants) were therefore excluded.

This narrowing of our inclusion criteria allowed a more detailed analysis of included trials, including the extraction and analysis of contextual modifiers of effect as reported by the authors. The registered protocol stated that we had not finalised the data to be extracted, and that the following would be included:

- Setting: country and health facility
- Health worker: cadre, number of participants
- Intervention: describe (later categorise, e.g. written material, training workshop etc.), duration, cost
- Study type: e.g. RCT
- Method used to assess performance: describe (later categorise e.g. prescribing, observation etc.)
- Effect of intervention (including time after completion)

Contextual modifiers of intervention effect were added to this list once the inclusion criteria had been narrowed, as described. We followed the methodology for extraction and analysis as reported in the manuscript.
